# The imperative for cross-cultural medical education in globalized healthcare

**DOI:** 10.3389/fpsyg.2024.1326723

**Published:** 2024-07-25

**Authors:** Changiz Mohiyeddini

**Affiliations:** Department of Foundational Medical Studies, Oakland University William Beaumont School of Medicine, Oakland University, Rochester, MI, United States

**Keywords:** cross-cultural medical education, cultural awareness, health care, health equity, globalization

## Abstract

Current healthcare systems are like living creatures. They are highly complex, multi-faceted, and dynamic. They must constantly change and adapt; they are like a melting pot, brimming with both rich and diverse cultures from all corners of the world. Beyond just nationality, these systems can include many languages, religious beliefs, socioeconomic backgrounds, and unique health practices. The tides of globalization, multicultural societies, migration, and international collaboration are continuously reshaping how healthcare providers are educated and how healthcare is delivered in an equitable, inclusive, and fair manner. To keep pace with, and within, these highly vibrant socio-cultural frameworks, Cross-Cultural Medical Education is needed to educate healthcare professionals. This education is essential to create professionals who are not just skilled, but who are also culturally savvy and able provide fair and equal care to patients from all sorts of backgrounds. It provides professionals with foundational knowledge to navigate the complex landscape of diverse patient populations. Cross-Cultural Medical Education is thus of paramount importance to satisfy the need for effective cross-cultural communication and understanding in patient care preferences, ultimately leading to improved health outcomes.

Healthcare settings now mirror the rich and diverse cultures of the world, transcending geographical and cultural boundaries. Extending beyond just nationality, this diversity encompasses lingual diversity, religious beliefs, socioeconomic backgrounds, and health practices. The diversity in healthcare systems is fostered by complex, multi-faceted, and dynamic interplay between globalization, multicultural societies, migration, and international collaboration. A plethora of empirical studies delineates the impacts of sociocultural factors, race, and ethnicity on health and clinical care (Hill et al., 1990; Berger, 1998). Therefore, Cross-Cultural Medical Education is needed to ensure culturally competent and skilled healthcare professionals are available to provide equitable patient care to patients with diverse religious, ethnic, cultural, socioeconomic backgrounds, and perspectives. The unique health-related beliefs and norms of a patient are shaped by these factors. These factors shape traditions and habits that can significantly impact the recognition of symptoms, thresholds for seeking care, health behaviors, adherence to treatment and preventive measures and medications, and communication preferences (Denoba et al., 1998; Betancourt et al., 1999; Coleman-Miller, 2000; Einbinder and Schulman, 2000; Flores, 2000; Gornick, 2000; Williams and Rucker, 2000). Consequently, sociocultural differences between patient and provider must be acknowledged and addressed in medical education to provide future physicians the skills and knowledge to successfully navigate the complex landscape of diverse patient populations and to be able to provide effective care to patients.

Failing to address these cultural differences can lead to misunderstandings, medical errors including misdiagnoses, and suboptimal treatment outcomes. Empirical evidence shows that patient dissatisfaction, distrust, poor adherence, and poorer health outcomes are most likely to occur when the cultural diversity of patients and providers are ignored and not communicated during the medical encounter (Betancourt et al., 1999; Cooper-Patrick et al., 1999; Langer, 1999; Morales et al., 1999; Stewart et al., 1999; Flores, 2000). Patient satisfaction, adherence to treatment, and overall health outcomes are improved with culturally tailored care that respects patients’ cultural values and beliefs (Betancourt et al., 2003; Beach et al., 2005).

According to Betancourt (2003), three key factors inspired the emergence of Cross-Cultural Medical Education: The need for preparing providers to be able to provide healthcare for a growing, diverse population (Zweifler and Gonzalez, 1998), to help to eliminate the pervasive racial/ethnic disparities in medical care seen today 9, and the standards put forward by accreditation bodies for medical training (i.e., the Liaison Committee on Medical Education) that require cross-cultural curricula as part of undergraduate medical education (Liaison Committee on Medical Education, n.d.).

There is another important issue that must be addressed that underscores the necessity of Cross-Cultural Medical Education in addition to these three factors. It is the recognition that healthcare providers themselves bring their own cultural backgrounds into their encounters with the healthcare system, with their colleagues, and with their patients (Betancourt, 2003). This aspect of cultural transaction is often overlooked, however, providers’ personal beliefs, values, and biases can significantly influence their interactions and decision-making processes. As such, current cross-cultural education must be extended to comprehensively address these influences. In other words, it is not only the culture of the patient that matters; the provider’s “culture” is equally important. Historical factors for patient mistrust, provider bias, and their impacts on physicians’ decision-making have also been documented (Betancourt, 2003). Failure to take sociocultural factors into account may lead to stereotyping, and, in the worst cases, biased or discriminatory treatment of patients based on race, culture, language proficiency, or social status. Training that encourages self-reflection and awareness of one’s own cultural identity can be incorporated to ensure healthcare providers can become more empathetic and effective in their practice. This approach involves exercises and techniques that promote self-reflection, including understanding one’s culture, biases, tendency to stereotype, and appreciation for diverse health values, beliefs, and behaviors. Examples from this training include having open conversations exploring the impacts of racism, classism, sexism, homophobia, and other types of discrimination in healthcare; determining whether providers have ever dealt with feeling “different” in some way and how they have dealt with that; attempting to identify—using patient descriptors or vignettes—hidden biases the student may have based on subconscious stereotypes; determining the student’s reaction to different visuals of patients of different races/ethnicities; and discussing ways in which individuals in the students’ families have interacted with the healthcare system (Betancourt, 2003; Betancourt et al., 2003; Liaison Committee on Medical Education, 2020; Liaison Committee on Medical Education, n.d.). By integrating these elements into Cross-Cultural Medical Education, future healthcare professionals can be better prepared to navigate their own cultural influences and those of their patients, leading to more equitable and effective healthcare outcomes.

Medical schools have adopted various approaches to integrate cross-cultural education into their curricula. For instance, these schools have dedicated standalone courses on cultural competence, covering topics such as cultural awareness, communication skills, and addressing health disparities among different ethnic and socioeconomic groups (Betancourt, 2003; Liaison Committee on Medical Education, 2020). Cultural content has also been integrated across various medical disciplines, to emphasize that cultural considerations are not isolated concepts, but they are integral to every facet of healthcare delivery (Betancourt et al., 2003). Another promising approach is the exposure of students to culturally diverse patient cases by utilizing simulation and case-based learning that allows students to engage in real-world scenarios involving diverse patients. This approach has shown it fosters critical thinking and empathy in students while they navigate complex cross-cultural dynamics. It encourages students to apply their cultural knowledge to make informed decisions and provide patient-centered care (Dogra et al., 2009).

However, while the integration of cross-cultural education into medical curricula has been deemed a necessity to foster healthcare delivery, the success of its implementation faces several challenges and barriers. First, well-validated and applicable faculty training and development are needed to adequately train and equip faculty members with the knowledge, skills, and attitudes required to aid students in navigating diverse cultural scenarios and to guide them through the complexities of culturally diverse and sensitive healthcare (Dogra et al., 2009). This need calls for comprehensive faculty development programs that emphasize cultural awareness, communication skills, and an understanding of the psycho-social determinants of health affecting diverse populations (Betancourt, 2003). Second, developing and implementing cross-cultural educational courses, materials, and training often requires additional financial resources, including funding for curriculum development and integration, cultural exchange experiences, and technology to facilitate cross-cultural interactions. Third, the integration of cross-cultural education may cause resistance to change within academic institutions. Faculty members, administrators, and even students may harbor implicit cultural biases that can impede their willingness to acknowledge and appreciate diverse values, norms, or perspectives. A multifaceted approach is required to addressing these biases, including educational campaigns to raise awareness, diversity and inclusion training, and fostering open dialogues and exchanges about cultural stereotypes and prejudices (Chapman et al., 2018). Finally, the development of valid and reliable assessment tools that capture the nuances of cultural competence is an essential companion to this infusion of cross-cultural education. Traditional assessment methods, such as written exams, may not effectively evaluate a student’s ability to interact with culturally diverse patients in real-world healthcare settings. Objective structured clinical examinations with cross-cultural scenarios, standardized patient interactions, and reflective portfolios are some strategies employed to evaluate students’ cultural sensitivity, communication skills, and patient-centered care (Luo et al., 2021).

Addressing these challenges are key to further enhancing the effectiveness of Cross-Cultural Medical Education. As healthcare systems evolve so must cross-cultural medical education. Therefore, faculty development programs should include interactive workshops, seminars, and cultural immersion experiences to keep paste with changes in the sociocultural environment for healthcare systems and to deepen educators’ understanding of diverse patient backgrounds and adjust and improve their teaching methods. Certainly, the economic burden of educating culturally aware and sensitive educators must be addressed by pursuing financial support to be sought through grants, partnerships with cultural organizations, and government funding. Academic institutions should address and overcome resistance to change by implementing continuous diversity and inclusion training, along with creating platforms for open dialogue and discussions on implicit biases and cultural stereotypes. Innovative assessment tools that go beyond traditional methods should be developed, including technology-driven simulations and real-time feedback from culturally diverse standardized patients.

Despite these challenges, the field of healthcare will continue to be confronted with a pressing need to adapt and ensure equitable patient care for individuals from numerous backgrounds. It is essential for healthcare professionals to be proficient in culturally sensitive care to navigate the complex landscape of diverse patient populations. Medical educators must remain committed to incorporating feedback, evolving best practices, and innovative teaching strategies to ensure that future healthcare professionals are well-equipped to provide high-quality, culturally competent care to all patients, thereby improving overall health outcomes and achieving health equity. Cross-Cultural Medical Education has now become of paramount importance, an imperative, to satisfy the need for effective cross-cultural communication and understanding in patient care.

## Data availability statement

The original contributions presented in the study are included in the article/supplementary material, further inquiries can be directed to the corresponding author.

## Author contributions

CM: Conceptualization, Writing – original draft.
